# Comparison of BHK-21 cell growth on microcarriers vs in suspension at 2L scale both in conventional bioreactor and single-use bioreactor (Univessel^® ^SU)

**DOI:** 10.1186/1753-6561-7-S6-P40

**Published:** 2013-12-04

**Authors:** Lídia Garcia, Elisenda Viaplana, Alicia Urniza

**Affiliations:** 1 Zoetis Manufacturung & Research Spain, S.L Pfizer Olot S.L.U., Ctra. Camprodon s/n, La Riba, 17813 Vall de Bianya (Girona), Spain

## Background

BHK-21 cells are the most commonly used cells for vaccine production. Not all cell lines can be adapted to suspension growth. In general, anchorage-dependent cells (must be attached to a substrate to grow) will grow in suspension only with the use of microcarrier beads. However, some cell lines such as the BHK-21 can be adapted to grow in suspension.

In recent years, the use of disposables in the pharmaceutical industry has increased extensively. The aim of this study is to evaluate the influence of a single use bioreactor on the final cell production of BHK-21 cells when they are growing with microcarriers or in suspension which can do an impact on the final product quality.

Cultivations on conventional 2L-bioreactors were compared with results obtained from 2L single use bioreactor (UniVessel^® ^SU).

## Materials and methods

### Cell line

Two BHK-21 cell lines were used, BHK-21 clone C3 as an anchorage-dependent cell line and SBHK cells adapted to grow in suspension.

Both cell lines were cultivated in MEM Glasgow medium supplemented with fetal bovine.

BHK-21 cells were grown in microcarriers Cytodex-3.

### Cultivation system

The growth using two different bioreactors was analyzed: Conventional reusable bioreactor (Autoclaving glass vessel of 2L ) and the UniVessel^® ^SU as a single use bioreactor

To control both bioreactors the BIOSTAT^® ^B plus unit was used.

Parameters as pH, temperature, stirring speed, aeration rate and viable cell number were analyzed.

Cell growth was conducted at the optimal conditions determined previously on spinner flasks. Cells were seeded into the bioreactor at the following concentration:

BHK-21: 5 × 10^5 ^cells/ml with a viability of ≥ 98%

SBHK: 3 × 10^5 ^cells/ml with a viability of ≥ 97%

### Cell count

BHK-21 were counted using the crystal violet dye nucleus staining method.

SBHK cells were counted using the NucleoCounter (ChemoMetec A/S).

## Results

Optimization, characterization of BHK cells culture processes and evaluation of microcarriers vs non-microcarrier processes at 2L scale were done.

Process performance was compared in conventional glass vessels to single use bioreactors.

In Table 1 values of viability and final cell density are shown in single-use and conventional bioreactors (3 batches per bioreactor). The results obtained demonstrated that at 3 days of culture no significant differences were found using both bioreactors.

**Table 1:** Cell growth and viability at 3 days culture in a 2L conventional bioreactor and in a single use bioreactor

	Univessel SU (2L)				Conventional Bioreactor (2L)			
	BHK-21		SBHK		BHK-21		SBHK	
Batch	Viable Cells (cells/ml)	Viability (%)	Viable Cells (cells/ml)	Viability (%)	Viable Cells (cells/ml)	Viability (%)	Viable Cells (cells/ml)	Viability (%)
**1**	2.90 × 106	97	2.70 × 106	99.1	2.46 × 106	99	1.96 × 106	92.1
**2**	1.20 × 106	96.5	1.95 × 106	90.5	1.80 × 106	98.9	2.36 × 106	98
**3**	2.10 × 106	98	3.0 × 106	97	1.90 × 106	98.1	3.50 × 106	99.4
**Mean valors**	2.08 × 10^6^	97.5	2.55 × 10^6^	97	2.05 × 10^6^	99	2.61 × 10^6^	99.4

**Figure 1:**
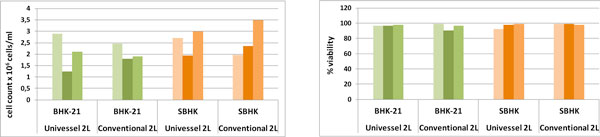
**Comparison of cell growth and viability at 3 days culture in a 2L conventional bioreactor and in a single use bioreactor**.

BHK-21 attached and grew efficiently on microcarriers. Fully confluency and a maximum viable cell density (between 1.2 to 2.9 × 10^6 ^cells/ml) was obtained after 3 days of culture (Table 1, Figure 1). In all the cases, the viability was higher than 96.5%.

SBHK cells reached higher yields comparing with the BHK-21. The maximum viable cell density (> 90% of viability) was obtained at 3 days of culture reaching a cell concentration between 1.95 to 3.5 × 10^6 ^cells/ml (Table 1, Figure 1).

The variability on final cell density obtained between the different batches was similar in both types of bioreactors (Table 1).

## Conclusions

✓ Comparable results between conventional glass vessels and single use bioreactors: cell density and viability.

✓ Given the good results obtained with SBHK cells, elimination of microcarriers can decrease the cost of a large-scale operation.

✓ The feasibility of transferring the BHK cells growth from a conventional bioreactor to single-use bioreactor has been demonstrated.

✓ Benefits of single-use technology integration:

• SU Bioreactors can replace conventional bioreactors without loss of process efficiency

• The scale-up for both suspension and attached cell lines in SU bioreactors is guarantee. The flexibility and easy of use of this SU bioreactors enable rapid scale-up without any loss in product quality

• SU Bioreactors increase easy of handling and offer advantages in the areas of cleaning, sterilization, validation, set-up, and turn-around time between runs.

• SU Bioreactors are the best solution when containment is required (BL-3 and BL-4 laboratories).

